# Resistance Training in Hypoxia as a New Therapeutic Modality for Sarcopenia—A Narrative Review

**DOI:** 10.3390/life11020106

**Published:** 2021-01-30

**Authors:** Won-Sang Jung, Sung-Woo Kim, Jeong-Weon Kim, Hun-Young Park

**Affiliations:** 1 Physical Activity and Performance Institute, Konkuk University, 120 Neungdong-ro, Gwangjin-gu, Seoul 05029, Korea; jws1197@konkuk.ac.kr (W.-S.J.); kswrha@konkuk.ac.kr (S.-W.K.); 2 Major in Exercise Therapy, Graduate School of Professional Therapy, Gachon University, 1342 Seongnam-daero, Sujeong-gu, Seongnam-si 13120, Gyeonggi-do, Korea; zeezone@gachon.ac.kr; 3 Department of Sports Medicine and Science, Graduate School, Konkuk University, 120 Neungdong-ro, Gwangjin-gu, Seoul 05029, Korea

**Keywords:** resistance training in hypoxia, sarcopenia, muscular function, hypertrophy, new therapeutic modality

## Abstract

Hypoxic training is believed to be generally useful for improving exercise performance in various athletes. Nowadays, exercise intervention in hypoxia is recognized as a new therapeutic modality for health promotion and disease prevention or treatment based on the lower mortality and prevalence of people living in high-altitude environments than those living in low-altitude environments. Recently, resistance training in hypoxia (RTH), a new therapeutic modality combining hypoxia and resistance exercise, has been attempted to improve muscle hypertrophy and muscle function. RTH is known to induce greater muscle size, lean mass, increased muscle strength and endurance, bodily function, and angiogenesis of skeletal muscles than traditional resistance exercise. Therefore, we examined previous studies to understand the clinical and physiological aspects of sarcopenia and RTH for muscular function and hypertrophy. However, few investigations have examined the combined effects of hypoxic stress and resistance exercise, and as such, it is difficult to make recommendations for implementing universal RTH programs for sarcopenia based on current understanding. It should also be acknowledged that a number of mechanisms proposed to facilitate the augmented response to RTH remain poorly understood, particularly the role of metabolic, hormonal, and intracellular signaling pathways. Further RTH intervention studies considering various exercise parameters (e.g., load, recovery time between sets, hypoxic dose, and intervention period) are strongly recommended to reinforce knowledge about the adaptational processes and the effects of this type of resistance training for sarcopenia in older people.

## 1. Introduction

Hypoxia refers to a condition in which oxygen saturation of arterial blood decreases and the amount of oxygen supplied to tissues is reduced, and is used for altitude/low oxygen training in natural or simulated high oxygen environments [1,2]. The hypoxic condition is a combination of a decrease in atmospheric pressure and a decrease in oxygen fraction, which can be divided into hypobaric hypoxia and normobaric hypoxia in general. Hypobaric hypoxic condition refers to a state in which both atmospheric and oxygen pressure decreases as the pressure decreases and the amount of air decreases, such as a natural high-altitude environment. The normobaric hypoxic condition refers to a low-oxygen environment in which the relative oxygen concentration is lowered by increasing the nitrogen concentration in the air by using nitrogen generating devices without pressure control [3]. Traditionally, training in altitude/hypoxic environments has been used to enhance oxygen transport capacity and oxygen utilization and improve sports performance through muscle oxygenation by high-intensity exercise and systemic desaturation by hypoxic conditions [4,5,6].

Nowadays, based on the lower mortality and prevalence of people living in high-altitude environments than those in low-altitude environments, hypoxia is being used to improve diabetes, cardiovascular diseases, hypertension, obesity, and age-related diseases [3]. Hypoxia is a new therapeutic modality for health promotion and disease prevention or treatment by body weight-related responses (e.g., decreased resting leptin level, increased adrenergic system, increased resting norepinephrine, increased blood serotonin levels, and suppressed appetite), cellular metabolic responses (e.g., hypoxic inducible factor-1α and vascular endothelial growth factor (VEGF) expression, angiogenesis, increased glycolytic enzymes and number of mitochondria, improved insulin sensitivity, and increased glucose transporter-4), cardiovascular responses (e.g., increased peripheral vasodilation, increased diameter of arterioles, increased oxygen affinity for hemoglobin, normalizes blood pressure, and cardiovascular protection), and respiratory responses (hyperventilation, increases lung diffusion, increases carbon dioxide excretion reserve during sleep, decreases arterial oxygen saturation, increases ventilation during exercise, and improves respiratory function) [7,8,9]. Hypoxia increases oxygen consumption and metabolism when exposed to the environment more than 2500 m (fraction of inspired oxygen; F_I_O_2_ < 15%) compared to sea level, and effectively increases energy consumption of various movements, such as low intensity intermittent and aerobic exercise [1,10]. Overweight and obese patients can achieve higher metabolic demands and lower walking speed is also likely more protective of the muscles/joints in obese patients with orthopedic comorbidities [11,12]. As a result, exercise in a hypoxic environment can improve weight loss, metabolism, and cardiopulmonary health, despite the low load and low mechanical strain [7,8,9,11,12,13].

Recently, resistance training in hypoxia (RTH), a new therapeutic modality combining hypoxia and resistance exercise, has been attempted to improve muscle hypertrophy and muscle function [14,15,16,17]. In particular, RTH induces greater muscle size, strength, endurance, and angiogenesis in the skeletal muscles [11,15,18,19]. Moreover, RTH with moderate intensity and high repetition is effective in improving the neuroendocrine responses, skeletal muscle fiber cross-sectional area (FCSA), lean body mass, muscle strength, muscle endurance, and physical function [11,14,16,17,20,21,22,23]. This suggests that RTH can be used as an effective therapeutic modality for improving sarcopenia, which is defined as the involuntary loss of skeletal muscle mass and function due to aging. In other words, RTH may play a role in slowing sarcopenia development as well as improving physical function via hypotensive and antioxidant actions and the well-being of elderly individuals [11,13,24].

Therefore, this review would like to explain the possibility of RTH with low exercise intensity and mechanical tension as a novel and valuable treatment that can be effectively applied to the sarcopenia population, along with recent previous studies. We can discuss the clinical and physiological aspects of sarcopenia and RTH for muscular function and hypertrophy.

## 2. Clinical and Physiological Point in Sarcopenia

Involuntary loss of skeletal muscle mass and function, defined as sarcopenia, is one of the most notable corollaries of aging and is a predictor of physical disability/mortality [25,26]. Sarcopenia affects nearly one-third of the aging population [27] and is one of the major contributors to negative health outcomes in older people [28]. There are several methods for diagnosing sarcopenia, but this is mainly determined by a calculation formula that divides the appendicular skeletal muscle mass measured by dual energy X-ray absorptiometry by the square value of height (men: ≤7.26 and women: ≤5.45) [29,30].

In people with an inactive lifestyle, muscle mass loss usually progresses gradually from age 40 and accelerates after age 60, resulting in a loss of approximately 2% of muscle mass per year [31]. Muscle strength decreases faster than muscle mass and is known to decrease by 3% per year from age 60 and by up to 40% per 10 years after age 70 [32,33]. Loss of muscle mass due to aging results in a decrease in fatty acid mobilization and an increase in body fat mass and, consequently, has a negative effect on various metabolic diseases mediated by obesity [34], and it also affects blood lipids, inflammation response, and production of metabolic hormones [35,36,37,38]. In the elderly, a decrease in muscle strength due to loss of muscle mass increases the risk of falls by 2–3 times [39,40]. In addition, elderly people with sarcopenia have a fall risk of approximately 2.6 times higher than that of normal elderly people [29], while elderly people with low muscle mass and muscle strength had 1.4 times and 2.34 times higher mortality rates, respectively [41]. Therefore, the loss of muscle mass causes a very serious problem in daily life and quality of life of the elderly [42,43].

**Figure 1:**
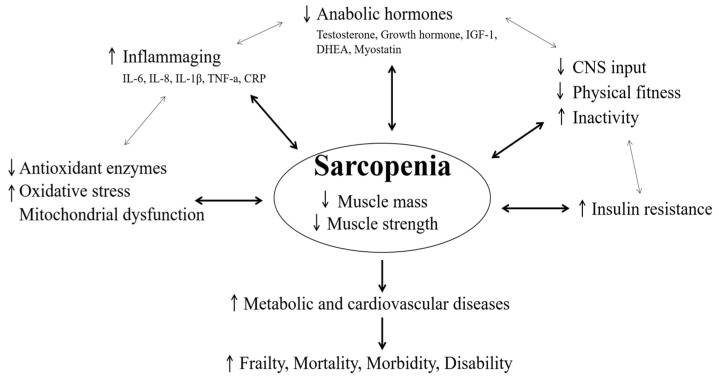
The mechanism for various clinical and physiological points related to sarcopenia. IL = interleukin; TNF = tumor necrosis factor; CRP = C-reactive protein; IGF-1 = insulin-like growth factor-1; DHEA = dehydroepiandrosterone; CNS = central nervous system.

In this section, various clinical and physiological points related to sarcopenia are described in relation to physical fitness, inflammation responses, antioxidants, and mitochondrial function, hormones, biochemical properties of muscles, and metabolic and cardiovascular diseases. The mechanism for various clinical and physiological points related to sarcopenia is shown in Figure 1.

### 2.1. Physical Fitness

The decrease in muscle mass in the process of degeneration of the human body is due to the decrease in the number and FCSA [44]. This phenomenon attenuates muscle strength as well as the muscle volume and causes a decrease in muscle endurance by a combination of two factors of muscle strength and muscle volume [45]. Sarcopenia affects physical functions such as cardiopulmonary function, flexibility, balance, and speed, which are affected by the musculoskeletal system, making it difficult for the elderly to live independently [46,47,48,49]. Therefore, elderly people with sarcopenia are more severely attenuated in physical fitness and function than the general population of elderly people, and it is known that the impairment of walking ability and the decrease in balance ability are severe [39,50,51,52]. The impairment of walking and balance ability is a direct cause of falls, and because falls account for the second cause of mortality in the elderly, physical fitness and function decline caused by sarcopenia is a very important factor in the quality of life [28,53,54]. Moreover, in humans, as muscle mass accounts for approximately 30% of the basal metabolism rate, a decrease in muscle mass acts as a direct cause of obesity and obesity-related diseases [35,55,56]. Therefore, sarcopenia is the cause of lowering physical strength and function, and it can be linked to causing obesity and obesity-related diseases by making the body composition state negative by reducing energy consumption.

### 2.2. Inflammation Responses

Decrease in muscle strength and obesity in the elderly are highly correlated with chronic inflammatory conditions [57]. de Luca and Olefsky [58] reported that an inflammatory condition occurs because macrophages are concentrated around adipocytes due to sarcopenic obesity, and macrophages induce both autologous and endocrine inflammatory signals around adipocytes. In previous studies on sarcopenia and inflammation, interleukin (IL)-6 and C-reactive protein, which are representative inflammatory cytokines, showed a positive correlation with total fat mass and a negative correlation with muscle mass [59,60]. Additionally, Visser et al. [40] also reported that there was a negative correlation between serum IL-6 or tumor necrosis factor-α levels and muscle mass. Therefore, the inflammatory level is very important in sarcopenia because inflammatory cytokines promote the breakdown of myofibrillar protein and reduce protein synthesis, causing a decrease in muscle mass [61,62].

### 2.3. Antioxidants and Mitochondrial Function

As one of the important causes of metabolic abnormalities associated with sarcopenia, reactive oxygen species produced in tissues are receiving much attention [43]. Free radicals produce peroxide, which damages and transforms DNA and destroys cells, thereby promoting aging and causing various diseases [43,63]. Cerullo et al. [64] reported that the major cause of muscle loss caused by aging is cell damage caused by oxidative stress. Moreover, it has been reported that oxidative stress caused by free radicals impairs the function of mitochondria in cells, thereby reducing muscle function and accelerating sarcopenia [65,66]. Currently, it has been largely demonstrated that the regulation of mitochondrial function can lead to the death of senescent cells and that removing senescent cells improves musculoskeletal health, quality, and function [67,68,69]. Ferri et al. [43] also reported that mitochondrial dysfunction in skeletal myocytes caused by free radicals is recognized as a major driver of sarcopenia. This indicates that the antioxidant ability to resist oxidative stress is an important factor in preventing sarcopenia in the elderly.

### 2.4. Hormones

Aging is known to cause changes in the expression and sensitivity of growth hormones (GH), insulin-like growth factor (IGF)-1, cortisol, testosterone, and female hormones [70,71,72]. These hormones are very important factors in understanding the metabolic state of sarcopenia because they affect the anabolism and catabolism of muscles [73]. IGF-1 is known to be an independent risk factor for sarcopenia, and a decrease in GH, estrogen, and dehydro-epiandrosterones, a precursor of testosterone, is known to be the cause of sarcopenia [74]. In addition, a remarkable decrease in the secretion of GH due to aging adversely affects body composition, resulting in worsening physical fitness and function [75,76,77]. Therefore, it can be seen that the decrease in the amount of secretion of various anabolic hormones due to aging is a physiological condition that cannot be overlooked because it is a major cause of worsening sarcopenia.

### 2.5. Biochemical Properties of Muscles

In the balance between protein synthesis and degradation for the maintenance of skeletal muscle tissue, the activated phosphatidylinositol-3-kinase (PI3K)/Akt/mTOR signaling pathway plays an important role in anabolic metabolism induced by protein synthesis [78,79]. Increased serum insulin and IGF-1 levels inhibit the suppression of muscle synthesis by activating the phosphorylation of insulin receptor substrate 1 and inducing the activation of the PI3K/Akt pathway and stimulation of mTOR, resulting in muscle hypertrophy [80,81,82]. In addition, serum insulin and IGF-1 levels influence the regulation of skeletal muscle FoxO proteins, members of the family of Forkhead transcription factors [83]. The FoxO family consisting of FoxO1, FoxO3, and FoxO4 is located in the nucleus and inhibits the PI-3K/Akt and mTOR signaling pathways to reduce the number and regeneration of satellite cells, thereby inducing sarcopenia [84,85].

Myostatin is a muscle growth regulatory factor produced by skeletal muscle and is regulated by the transcription factors for small mothers against decapentaplegic (SMAD) 2 and 3, which induce IGF-1/Akt signaling [86]. Myostatin induces muscle atrophy by upregulating ubiquitin ligases, atrogin1, and muscle RING-finger protein-1 (MuRF1) via FoxO transcription factors [87]. Moreover, MAPK, a Ser/Thr kinase, regulates extracellular signals involved in a wide range of cellular processes such as gene expression, apoptosis, and differentiation in eukaryotic cells, and an upstream of MAPK stimulates the phosphorylation of tyrosine and threonine residues after receiving signals from cytokines, growth factors, and cellular stressors, resulting in the activation of MAPKs [88,89].

Consequently, it can be explained that these various biochemical pathways regulate protein synthesis and degradation, thereby affecting sarcopenia.

### 2.6. Metabolic and Cardiovascular Diseases

Sarcopenia reduces daily energy consumption, leading to obesity and type 2 diabetes [90,91,92]. Obesity and type 2 diabetes show a high positive correlation, and the deposition of intramuscular triglyceride induced by sarcopenia causes insulin resistance [93,94,95]. Insulin is a hormone that causes muscle anabolic action, but when insulin resistance occurs due to obesity, it acts as a factor that promotes muscle catabolism, and worsens sarcopenia [96].

Additionally, the loss of muscle mass with increasing age induces obesity, type 2 diabetes, inflammation, and oxidative stress, which in turn increases the risk of cardiovascular disease [97,98,99,100]. Sarcopenia accompanying aging is primarily due to a decrease in the fast-twitch FCSA, which subsequently leads to accumulation of body fat and reduces vascularization and angiogenic capacity, and consequently increases the risk of cardio-metabolic disorders [11,101,102].

## 3. Resistance Training in Hypoxia for Muscular Function and Hypertrophy

Exercise intervention is the most commonly used method to improve sarcopenia [103]. Among the methods used to improve sarcopenia, resistance training is reported to be effective in improving muscle mass and muscle function [104,105]. Resistance training is effective in treating and preventing sarcopenia by modulating muscle protein synthesis to improve muscle strength and skeletal muscle mass [103,104]. Moreover, resistance training not only improves muscle mass and function but also lowers insulin sensitivity, glycated hemoglobin, and blood inflammation and increases bone mineral density in the elderly [106,107,108,109,110,111,112,113]. Verdijk et al. [114] reported that 80% of one repetition maximum (1-RM) of resistance training for 12-week (3 times a week) improved femoral muscle area and strength in the elderly aged 65 to 85 years. A study by Kryger and Andersen [115], which was conducted with the same duration and intensity as Verdijk et al. [114], also reported an increase in the size of type II fibers and muscle strength by resistance training. As such, a high exercise load is required for remarkable muscle mass and muscle function improvement by resistance training. In general, it is known that the optimal exercise intensity for resistance exercise to improve muscle mass and muscle function is at least 70% to 80% of 1-RM [114,115,116]. 

However, such high intensity can cause problems with the musculoskeleton for inactive elderly people to perform high-intensity resistance training to slow the development of sarcopenia and improve muscle function [13,103]. In consideration of stability, several previous studies that performed resistance training at a relatively low intensity reported that there was no effect on muscle size, muscle strength, and functional capacity in sarcopenic elderly population [117,118]. To overcome these shortcomings, low-intensity resistance training with blood flow restriction, which induces local hypoxia, has been widely used, but this method has the disadvantage of excessive blood pressure during exercise [119]. Therefore, resistance training in systemic hypoxia, that is, RTH, which positively affects the parameters of physical ability and body composition over life, has been widely used in recent years [24]. Interestingly, RTH has been reported to be more effective in improving FCSA, lean body mass, and muscle function with moderate intensity and many repetitions than with high intensity [15,16,20]. Of course, it cannot be definitively argued that RTH brings great advantages over traditional resistance exercise, Camacho-Cardenosa et al. [120] investigated the therapeutic benefits of 18-week of whole-body vibration training (WBVT) under hypoxia equivalent to 0.16 F_I_O_2_ on the strength function of an elderly population. As a result, the WBVT showed a significant enhancement in the maximal strength of knee extensors with a small effect size. They concluded the WBVT under hypoxia did not have a remarkable effect on strength function in healthy elderly subjects. Like this, it cannot be definitively argued that RTH brings great advantages over traditional resistance exercise, but it is considered to have a great advantage in envisioning a more efficient and effective training strategy. Therefore, it is very important to interpret the effect of RTH on muscle function and hypertrophy as a new therapeutic modality for sarcopenia.

### 3.1. Effect of Resistance Training in Hypoxia on Muscle Morphological and Function Change

First, with regard to the morphological change via RTH, Manimmanakorn et al. [20] designed the RTH program to perform 20% 1-RM of resistance training for 5 weeks to female netball players under a hypoxia equivalent to 80% of the peripheral oxygen saturation (S_p_O_2_). As a result, the RTH group showed a 3.2% increase in the sum of the FCSA of the knee flexors and flexors compared to the traditional resistance training group. Nishimura et al. [121] reported a significant increase in FCSA in the RTH group as a result of applying 70% 1-RM resistance exercise in hypoxia (using vascular occlusion) for 6 weeks to healthy men. However, Friedmann et al. [122] reported no significant increase in FCSA when healthy men performed 30% 1-RM resistance training under hypoxic conditions (F_I_O_2_ = 0.12) for 4-week.

These discrepancies can be explained by differences in RTH program duration, intensity, repetition, and rest between sets [19,123]. Manimmanakorn et al. [20] and Nishimura et al. [121] conducted the study for 5 and 6 weeks, respectively, while Friedmann et al. [122] performed the study for 4 weeks. Moreover, Nishimura et al. [121] reported positive changes in FCSA through moderate exercise intensity (70% 1-RM), while Friedmann et al. [122] considered that there was no change in FCSA because an RTH of too low intensity (20% 1-RM) was performed. However, Manimmanakorn et al. [20] reported positive results for FCSA, even though RTH was performed with low-intensity exercise (20% 1-RM). This result is considered to be the difference in recovery time between sets (Friedmann et al. [122]: 60 s recovery vs. Manimmanakorn et al. [20]: 30 s recovery). From these results, brief recovery time periods in low-intensity RTH program appear to be a sufficient time to remove various metabolites produced by exercise. Consequently, it is thought that FCSA did not increase because it did not induce metabolic and hormonal responses by metabolites. In other words, a low-intensity RTH program for increasing muscle mass and FCSA in the elderly is considered as potentially desirable to provide a short recovery time between sets.

RTH has been reported to be effective in improving muscle functions such as muscle strength and endurance, as well as muscle morphological changes such as FCSA [15,17,20,121]. Kon et al. [15] divided 16 healthy males into a control group (*n* = 7) that performed exercise resistance under normoxia and an experimental group (*n* = 9) that conducted exercise resistance under hypoxia (F_I_O_2_ = 14.4%), and then all subjects performed 8 weeks of resistance training in each environmental condition. Consequently, the experimental group that performed RTH showed a significant improvement in muscle endurance, but no difference in muscle strength. Park and Lim [17] verified the effect of RHT after equally dividing competitive swimmers into a normoxic training group (*n* = 10) and a hypoxic group (*n* = 10) for training at 526 mmHg hypobaric hypoxic condition. They reported a greater improvement in muscle function (e.g., muscular strength and endurance) and anabolic hormone (e.g., GH, IGF-1, and VEGF) in the hypoxic training group than in the normoxic training group. Further, Manimmanakorn et al. [20] designed the RTH program to perform 20% 1-RM of resistance training for 5 weeks to female netball players under hypoxia equivalent to 80% of the peripheral oxygen saturation (S_p_O_2_). Consequently, the RTH group showed significantly improved muscle strength and endurance than the traditional resistance training group. They also reported that the improvement of muscle functions (e.g., muscle strength and endurance) via RTH can enhance skill-related fitness (e.g., agility). Törpel et al. [24] compared the effects of low- to moderate-load (25–40% of 1-RM, 3 × 15 repetitions) RTH (~80–85% S_p_O_2_) with matched designed resistance training under normoxia on normal muscular strength capacity and body composition after 5 weeks in young (*n* = 42) and older people (*n* = 42). They reported that the RTH design used had no superior effect on the tested parameters in young and older people over matched designed RT under normoxia after a 5-week intervention period. Based on this, they explained that it can be assumed that the expected higher effect of RTH can be achieved by changing the exercise parameters (e.g., longer intervention period and higher loads).

In summary, to utilize RTH as a new therapeutic modality for sarcopenia, further RTH intervention studies considering various exercise parameters (e.g., load, recovery time between sets, hypoxic dose, intervention period) are strongly recommended to reinforce knowledge about the adaptational processes and the effects of this type of resistance training in older people.

### 3.2. Potential Mechanism of Resistance Training in Hypoxia on Muscle Morphological and Function Change

**Figure 2:**
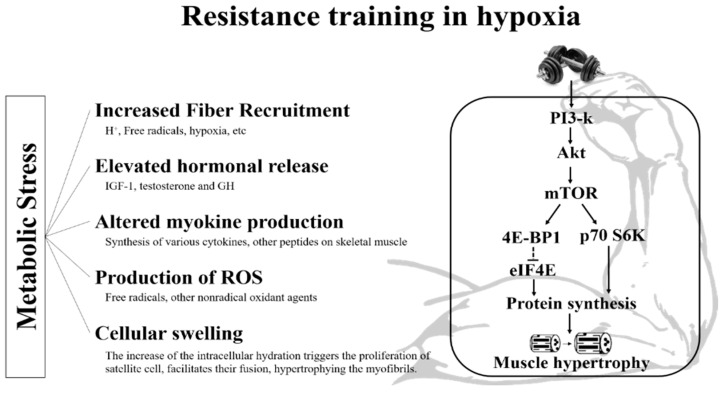
Proposed mechanisms with resistance exercises for hypoxia-induced metabolic stress may mediate muscular function and hypertrophy. ROS: reactive oxygen species. IGF-1 = insulin-like growth factor-1; GH = growth hormone; ROS = reactive oxygen species; PI3-k = Phosphoinositide 3-kinases; mTOR = mammalian target of rapamycin; 4E-BP1 = eukaryotic translation initiation factor 4E binding protein 1; eIF4E = eukaryotic translation initiation factor 4E; p70 S6K = 70 kDa ribosomal S6 kinase.

The most representative potential mechanisms for morphological changes (e.g., type II FCSA) and muscle function (e.g., muscular strength and endurance) via RTH are the accumulation of metabolites, hormonal response, and intramuscular signaling pathways. The proposed mechanism for muscle morphological changes and muscle function via RTH is shown in Figure 2.

The accumulation of metabolites has been reported to play various intervention roles in inducing muscle hypertrophy [124,125,126,127]. In particular, lactate, inorganic phosphate (Pi), arterial blood saturation, bicarbonate, and hydrogen ion (H^+^) are known metabolites that play a pivotal role in muscle hypertrophy [127,128,129]. Currently, many researchers believe that hypoxic stimulation affects contractile muscle proteins and plays a pivotal role in various stimulations for muscle hypertrophy, but not many studies have been conducted. Kon et al. [130] equally classified 12 active young men into an experimental group performing RTH (F_I_O_2_ = 0.13) and a control group performing resistance exercise in normoxia (F_I_O_2_ = 0.21). Then, the metabolic response immediately after performing bench press and leg press exercises with 10 repetitions for 5 sets with 70% 1-RM was examined. Consequently, the experimental group showed 1.2 times higher blood lactate level than the control group. Kon et al. [131] confirmed the metabolic response immediately after resistance exercise (14 repetitions, 5 sets, and 50% 1-RM) composed of bench press and leg press under normoxia and hypoxia in eight healthy men. As a result, lactate levels significantly increased after exercise in both trials, and the mean values of lactate were significantly higher in the HR trial than in the NR trial. Martínez-Guardado et al. [125] classified 25 untrained men into an RTH group (F_I_O_2_ = 0.13) and resistance exercise in normoxia group (F_I_O_2_ = 0.21). Then, they evaluated the acute effects of an RTH composed of 3 sets of 75% 1RM to muscle failure with a 2-min rest between sets to muscle failure on bench press performance. As a result, there was no difference in physical performance during bench press between the two groups, however, there were significant increases of blood lactate levels in the hypoxia group. Scott et al. [126] assessed whether moderate-load resistance exercise composed of 3 sets of 10 repetitions of squats and deadlifts at 60% of 1 repetition maximum with 60-s inter-set rest under hypoxia (F_I_O_2_ = 0.16) augments anabolic responses. As a result, they concluded moderate-load resistance exercise under hypoxia augments metabolite accumulation (e.g., increased blood lactate level and decreased arterial oxygen saturation) and muscle activation (e.g., electromyography). Ramos-Campo et al. [127] confirmed the effect of high-resistance circuit training composed of two blocks of three exercises (Block 1: bench press, deadlift and elbow flexion; Block 2: half-squat, triceps extension, and ankle extension) under hypoxia on metabolic and acid-base balance, blood oxygenation, electrolyte, and half-squat performance. They reported high-resistance circuit training under hypoxia showed an increased blood lactate level and decreased an arterial oxygen saturation, blood bicarbonate level, and pH. These previous studies suggest that the increase in metabolites such as blood lactate level via RTH is likely to improve muscle morphology and function. In the future, much research on this topic is desperately needed to use RTH as a therapeutic tool for sarcopenia.

Hormonal responses by RTH also play an important role in muscle morphological and functional changes [17]. In the studies by Kon et al. [130] and Kon et al. [131], significantly elevated GH and testosterone levels by low-(50% 1-RM) and moderate-intensity (70% 1-RM) resistance exercises in patients with hypoxia (F_I_O_2_ = 0.13) were reported. However, serum IGF-1 levels increased immediately after resistance exercise in both environmental conditions, but there was no difference between the two groups. These results suggest that elevated metabolic stress via RTH induces an increase in GH and testosterone [132,133]. Chycki et al. [22] evaluated the effect of 6-week RTH (F_I_O_2_ = 0.129) on hormonal response and muscle hypertrophy in resistance trained male subjects. Consequently, 6-week of RTH induced a significant increased IGF-1 level, body mass, and fat free mass compared to training in normoxia. In addition, Kon et al. [130] reported that metabolic hormones such as epinephrine, norepinephrine, and cortisol were significantly higher immediately after resistance exercise (70% 1-RM) in patients with hypoxia than in those with normoxia. However, Kon et al. [131] reported that there was no difference between the two environmental conditions in serum norepinephrine and cortisol levels when performing low-intensity resistance exercise equivalent to 50% 1-RM. The discrepancy between these two studies was observed to be due to the difference in resistance exercise load (low intensity: 50% 1-RM vs. moderate intensity; 70% 1-RM) and also because the higher the force required for muscle contraction, the higher the secretion rate of hormones such as catecholamine and cortisol [133,134,135]. Currently, the role of the systemic endocrine response in improving muscle function and hypertrophy is a point of debate among many scientists; therefore, it is very important to investigate the mechanisms by which various hormonal responses lead to improvement of muscle function and hypertrophy in future studies.

In the intramuscular signaling pathway, chronic exposure to hypoxia has been reported to negatively affect protein kinase B/mTOR signaling and increase myostatin expression, resulting in skeletal muscle atrophy [136,137,138]. However, the response to the intramuscular signaling pathway that occurs during resistance exercise after acute exposure to hypoxia is not yet clear. In the study by Etheridge et al. [139], seven healthy men (21.4 ± 0.7 years) performed unilateral leg resistance exercise (6 × 8 repetitions at 70% 1-repetition maximum) under normoxic (20.9% F_I_O_2_) and normobaric hypoxic (12% F_I_O_2_ for 3.5 h) post-absorptive conditions. They concluded that although the phosphorylation of ribosomal S6 kinase 1 was increased, hypoxia did not reduce muscle protein synthesis over 3.5 h at rest but blunted the increased muscle protein synthesis response to acute resistance exercise to a degree dependent on extant S_p_O_2_. Moreover, they suggested that other signaling processes that are probably not currently known in hypoxia could ignore mTOR (mechanistic target of rapamycin) signaling and trigger a physiological response to resistance exercise. Usually, exposure to hypoxia has been reported to activate hypoxic inducible factor-1α (HIF-1α), which acts as a primary transcriptional response factor for adaptation to hypoxia [140]. The activation of HIF-1α by exposure to hypoxia stimulates the expression of VEGF and increases angiogenesis [141,142,143]. Currently, it is reported that the expression of HIF-1α and VEGF has a positive effect on bone remodeling and repair, but its effectiveness in improving muscle function and hypertrophy is still unknown [144,145]. The response of skeletal muscle to RTH is affected by the upregulation of the autophagy-lysosomal pathway through a combination of metabolic and hypoxic stress [146]. This autophagy-lysosomal pathway is largely involved in the catabolic process in the atrophying muscle [147]. The signaling pathway by the autophagic process in skeletal muscle is reported to be very complex, but mTOR complex 1 can promote protein synthesis and autophagic processes [146,147]. In fact, it has been confirmed that muscle mass is maintained even though the protein degradation effect by the autophagy process is exhibited in skeletal muscle [146]. As a result of synthesizing previous studies, it seems that the role of hypoxic stimulation in both anabolic and catabolic processes is not yet clearly understood. Therefore, it can be a very interesting research topic to examine the mechanisms by which resistance exercise can influence the signaling pathway in muscles after acute exposure to hypoxia, and this is considered to be important basic research in using RTH as a new therapeutic modality for sarcopenia.

A recent Britto et al. [148] study reported that RTH promoted acute inflammation mediated by the TNFfi/NF-fiB/IL-6/STAT3 pathway, which contributed to myogenesis satellite cells. Inflammatory activation has been reported that CD68 and CD197 positive immune cells, known to control muscle regeneration, are activated in skeletal muscle and play a role in muscle regeneration. Therefore, RTH is expected to improve muscular dystrophy due to inflammatory reactions and immune activation of sarcopenia.

## 4. Conclusions

Resistance training provides a strong mechanical stimulus to the human body, through which metabolic changes, hormonal changes, and changes in intracellular signaling pathways appear, thereby inducing muscle hypertrophy and improving muscle function. However, traditional resistance training with high-intensity can cause problems with the musculoskeleton for inactive elderly people. Therefore, in recent years, RTH, which combines low- to moderate-intensity resistance exercise and hypoxia, that can induce physiological and biochemical changes related to muscle morphology and function more strongly has attracted much attention. However, there are very few studies that have applied RTH to the elderly with sarcopenia, and previous studies demonstrating that RTH has a greater effect on sarcopenia-related clinical and physiological points than traditional resistance training are also very scarce. Therefore, it is difficult to argue strongly that RTH is a more effective method for improving muscle function and hypertrophy in the elderly than traditional resistance training based on the review of previous studies. This is even more persuasive in that the potential mechanisms for the improvement of muscle function and hypertrophy that appear during RTH are not yet clearly identified. Further, RTH intervention studies considering various exercise parameters (e.g., load, recovery time between sets, hypoxic dose, and intervention period) are strongly recommended to reinforce knowledge about the adaptational processes and the effects of this type of resistance training in older people for utilizing RTH as a new therapeutic modality for sarcopenia.

## Data Availability

The study did not report any data.
